# Fatal stroke after completion pneumonectomy for torsion of left upper lobe following left lower lobectomy

**DOI:** 10.1186/1749-8090-1-25

**Published:** 2006-09-12

**Authors:** Efstratios Apostolakis, Efstratios N Koletsis, Nikolaos Panagopoulos, Christos Prokakis, Dimitrios Dougenis

**Affiliations:** 1 Department of Cardiothoracic Surgery, University of Patras School of Medicine, Greece

## Abstract

**Background:**

The lobar torsion after lung surgery is a rare complication with an incidence of 0.09 to 0.4 %. It may occur after twisting of the bronchovascular pedicle of the remaining lobe after lobectomy, usually on the right side. The 180-degree rotation of the pedicle produces an acute obstruction of the lobar bronchus (atelectasis) and of the lobar vessels as well. Without prompt treatment it progresses to lobar ischemia, pulmonary infarction and finally fatal gangrene.

**Case Presentation:**

A 62 years old female patient was admitted for surgical treatment of lung cancer. She underwent elective left lower lobectomy for squamous cell carcinoma (pT2 N0). The operation was unremarkable, and the patient was extubated in the operating room. After eight hours the patient established decrease of pO_2 _and chest x-ray showed atelectasis of the lower lobe. To establish diagnosis, bronchoscopy was performed, demonstrating obstructed left lobar bronchus. The patient was re-intubated, and admitted to the operating room where reopening of the thoracotomy was performed. Lobar torsion was diagnosed, with the diaphragmatic surface of the upper lobe facing in an anterosuperior orientation. A completion pneumonectomy was performed. At the end of the procedure the patient developed a right pupil dilatation, presumably due to a cerebral embolism. A subsequent brain angio-CT scan established the diagnosis. She died at the intensive care unit 26 days later.

**Conclusion:**

The thoracic surgeon should suspect this rare early postoperative complication after any thoracic operation in every patient with atelectasis of the neighboring lobe. High index of suspicion and prompt diagnosis may prevent catastrophic consequences, such as, infarction or gangrene of the pulmonary lobe. During thoracic operations, especially whenever the lung or lobe hilum is full mobilized, fixation of the remaining lobe may prevent this life threatening complication.

## Background

Lobar torsion after pulmonary lobectomy is a rare complication with an incidence of 0.09 to 0.4 % [1] and is developed after twisting of the bronchovascular pedicle of the remaining lobe after lobectomy, usually on the right side. The 180-degree rotation of the pedicle produces an acute obstruction of the lobar bronchus (atelectasis) and also of the lobar vessels. Without prompt treatment it progresses to lobar ischemia, pulmonary infarction and finally fatal gangrene [2,3].

In this paper we present a very rare case of fatal massive cerebral embolism, occurring immediately after re-operation for treatment of left-upper lobe torsion, complicating a left lower lobectomy.

## Case report

**Figure 1:**
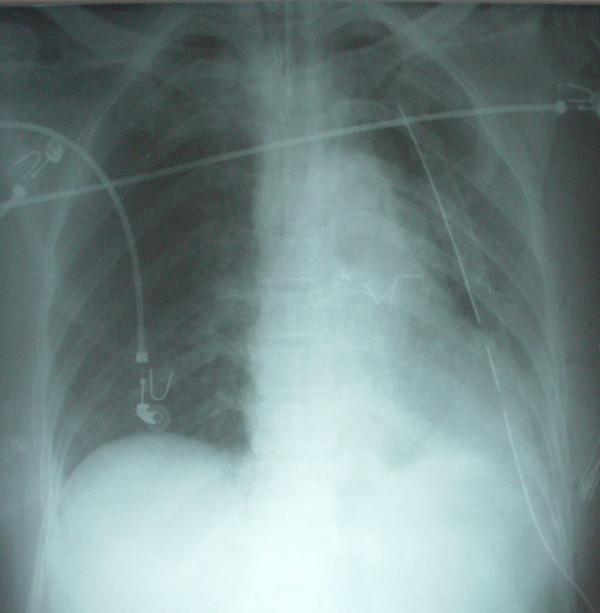
Early postoperative chest radiography showing a normal appearance.

**Figure 2:**
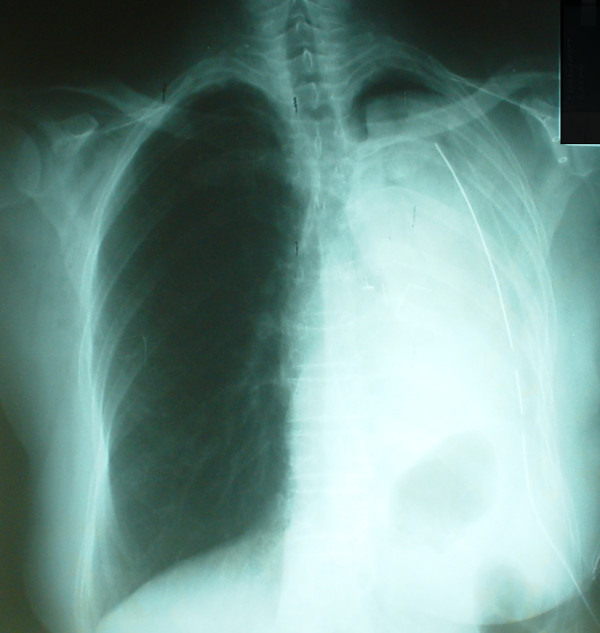
Chest X-ray 10 hours later with consolidation and loss of air entry.

A 62-year-old female patient, presented with a left lower lobe peripheral mass suspicious for malignancy. Flexible bronchoscopy was normal and fine needle aspiration cytology revealed a squamous cell carcinoma. Physical examination and routine laboratory data were unremarkable. Echocardiography was normal and FVC and FEV1 were 2.84 L and 2.45 L respectively. Chest CT scanning revealed no mediastinal lymphadenopathy. Additionally, brain and upper abdominal CTs scanning, as well as Technetium Tc 99 m bone scintigraphy, were all normal. The patient underwent a left lower lobectomy through a lateral thoracotomy and a rather peripheral tumour measuring 4 × 3 cm was found. No nodal involvement was observed macroscopically and this was confirmed with frozen sections of nodal sampling obtained from sites 5, 9, 10 and 11. Biopsy of the tumour proved to be a pT2N0, well differentiated, squamous cell carcinoma. Double lumen endotracheal tube and high thoracic epidural analgesia catheter for pain management were routinely used. No blood or blood products were transfused. The patient was extubated immediately postoperatively and the routine chest X-ray at the recovery room was reported normal with good expansion of the upper lobe with no residual pneumothorax (Fig. 1). Early postoperative course was uneventful. However, 8 hours later SpO_2 _pulse oximetry was noted to decline gradually, without any remarkable respiratory deterioration. Repeated chest radiography showed a left upper lobe consolidation (Fig. 2). Urgent bronchoscopy revealed occlusion of the lower left main bronchus; neither the left lower bronchial stump could be seen, nor the left upper bronchus could be entered. With a high index of suspicion of lobar torsion, the patient underwent urgent reoperation. Once the thoracic cavity was entered, the upper lobe was found to be ischemic and atelectatic with its diaphragmatic surface facing in an anterosuperior orientation. Just before clamping the hilum, the lobe was unfortunately re-orientated taking its proper position and immediately regained its physiological colour. The hilum was quickly clamped en-block. A completion pneumonectomy was easily carried out. At the end of the procedure, the patient developed a right pupil dilatation and it became obvious that a massive cerebral embolism had occurred. A subsequent brain angio CT scanning revealed total obstruction of the right middle cerebral artery, presumably due to emboli from the occluded pulmonary vein. The patient died at the intensive care unit 26 days later.

## Discussion

Lobar torsion is a very rare complication which may occur spontaneously, usually following pulmonary resections, or infrequently, after thoracic trauma [4,5] or any thoracic operation [6,7] including lung transplantation as recently reported [8].

Shamaun [4] reviewed 18 cases of postoperative lobar torsion and found 12 cases occurring after lung resections and 6 cases following other thoracic operations.

Wong and Goldstraw [9] and Shamaun [4] reported that the right middle lobe, after ipsilateral upper or lower lobectomy, is the most implicated lobe, but lobar torsion may also occur following any left lobectomy procedure. In a study of 7889 patients who underwent elective pulmonary resection, Cable [1] reported 7 cases of lobar torsion distributed in the right-middle lobe in two cases, the right-upper and middle in one, the right-lower in two and in the left-upper in another two cases.

Although the cause of this complication is not clear yet, it is believed that rotation or angulation of the lobar bronchus, results in pulmonary arterial and venous obstruction. The venous obstruction predisposes to gangrene of the lobe, but the time required for this to occur, is not well known. The coexisted obstruction of the bronchial arterial circulation produces lobar infarction [4].

Factors predisposing to lobar torsion are considered to be the following: a complete interlobar fissure, the absence of adhesions [10], a narrow middle lobe hilum [4] and an overzealous mobilization of the lobe after surgical disruptions of its intrathoracic attachments [11] including the routine division of inferior pulmonary ligament [12]. In the presence of a complete interlobar fissure, prevention of middle lobe torsion following upper lobectomy could be achieved by fixating the middle to the lower lobe with the use of TachoCombl, as recently described [13].

Early diagnosis is based on the high index of suspicion, which should be raised postoperatively when a patient suffers from: a) sudden and unexplained dyspnea, tachypnea refractory to oxygen supplementation, with or without deterioration of arterial blood gases, b) persistent high fever, hemoptysis and productive cough consisting of massive hemorrhagic or copious bronchial secretions [4,8,11]. More specifically, radiological signs in plain chest X-rays which could allow further confirmation of the suspicion of lobar torsion include: a) a sudden opacification and absence of expansion of the remaining and previously normal lobe, particularly if the opacificated lobe is in an unusual anatomic position, with or without a bronchial cut-off sign, b) the absence of mediastinal shift to the ipsilateral side and no intercostal narrowing [9,14]. Furthermore, if any early previous air leak ceases suddenly and if expansion of the collapsed lobe after vigorous tracheobronchial suction does not occur, although some copious and bloody secretions are aspirated, one should suspect the presence of lobar torsion and should proceed with a prompt bronchoscopy [15,16]. Bronchoscopy may reveal a compressed or occluded lobar bronchus appearing like a "fish mouth" [4]. It should be noted that although the instrument may be passed through the site of obstruction, the bronchus could collapse again after the bronchoscope is withdrawn [11]. Thoracic CT scanning, although not characteristic, it may help in establishing the diagnosis. It could show an increased opacification, occupying the remaining lobe. The absence of bronchogram in the consolidated area suggests the complete obstruction – "cut-off" sign – of the bronchus [17]. The site of rotation may be seen as obliteration [18]. Contrast-pulmonary arteriography during helical CT scanning has the advantage of direct evidence of stenosis or complete obstruction of kinking vessels [1,18,19]. The transesophageal echocardiography (TEE) may reveal the turbulent flow in the pulmonary vein and the presence of a potentially lethal thrombus. The lung-ventilation scan shows poor or no perfusion and minimal ventilation of the distorted lobe [14,20].

The benefits for a surgeon to be watchful, to diagnose and subsequently re-operate a patient with lobar torsion are the following: a) salvage of the lobe before it develops infarction or gangrene, especially for patients with critical preoperative pulmonary function unable to tolerate pneumonectomy, b) reduction of the risk of infection and sepsis, c) prevention of thrombosis in the obstructed pulmonary vein, which could be a catastrophic complication [14].

However, despite clinical and laboratory signs, lobar torsion may not be diagnosed early enough. According to the Mayo Clinic's experience, the median time for the diagnosis of a lobar torsion is 10 days postoperatively, ranging from 2 to 14 days [1]. Similarly, the two reported cases by Parambil were diagnosed 5 and 10 days after the initial operation [20].

In our case, a prompt diagnosis of this complication was definitely established and the patient underwent an emergency re-operation, approximately 16 hours after the initial thoracotomy. However, a thrombus had already been established in the occluded pulmonary veins.

It should be noted that, it was taken into consideration that during re-operation and untwisting of the lobe, to assess its viability, an already existing thrombus may be embolized. Our plan was to perform a completion pneumonectomy. However, just before clamping the hilum, the contorted lobe was suddenly untwisted to resume its physiological position (lobe repositioning), causing fatal cerebral embolism. Upon retrospective analysis of this case, we concluded that we should have reopened the thoracotomy carefully in a stepwise manner and meticulously keep the lobe in place until intrapericardial clamping of the pulmonary veins is placed.

Also, if a TEE had been done, pulmonary vein thrombus might have definitely been diagnosed prior to reoperation and therefore efforts could have been done to avoid untwisting.

To the best of our knowledge, only two cases of this rare complication have been described in the literature. The first-one was reported by Inoue et al. in 1990 [21]. They reported a 38-year old man, who, after a left lower lobectomy, had developed torsion of the upper lobe, on the 2^nd ^postoperative day. During re-operation, the patient developed right hemiplegia after repositioning of the upper lobe. The second-case referred to a patient who after an initial left upper lobectomy underwent a completion lobectomy of the contorted lower lobe. A massive cerebral infarction complicated the re-operation [22].

Treatment of lobar torsion consists of two options: either completion lobectomy or repositioning and fixation [4,10]. Sacrifice of the lobe is indicated whenever pulmonary infarction or gangrene has been suspected [4,5]. This is easily recognized upon inspection of the lobe which appears "blue-black". It is proposed that in any other case the lobe should be repositioned and fixated with a suture to the other lobe if it exists, or fixated with a pleural flap to the thoracic wall, to prevent torsion re-occurrence [4]. Other authors, especially for the delayed cases, recommend resection of the distorted lobe during re-operation, in order to avoid the possible complications of systemic embolism and/or septicemia, which may occur after repositioning of the lobe [8,14,15].

## Conclusion

In summary, we have described a case of left-upper lobe torsion following left-lower lobectomy. Despite prompt diagnosis, fatal cerebral emboli unfortunately occurred. In agreement with the literature available and our experience, we are of the opinion that in all re-operations, the surgeon should always keep in mind the possibility of pulmonary vein thrombosis and therefore, the lobe should be kept contorted intentionally, until pericardial clamping of the pulmonary veins has been applied. The lobe can be repositioned, and afterwards surgical decisions can be taken whether lobectomy should be performed, which, based on the accumulated knowledge, we consider the most appropriate procedure, or lobar salvage and fixation.

## List of abbreviations

FVC: Forced expiratory Vital Capacity

FEV1: Forced Expiratory Volume in one second

SpO_2_: Pulse Oximetry

## Competing interests

The author(s) declare that they have no competing interests.
